# Characterization of Structural Features Controlling the Receptiveness of Empty Class II MHC Molecules

**DOI:** 10.1371/journal.pone.0018662

**Published:** 2011-04-14

**Authors:** Bernd Rupp, Sebastian Günther, Talat Makhmoor, Andreas Schlundt, Katharina Dickhaut, Shashank Gupta, Iqbal Choudhary, Karl-Heinz Wiesmüller, Günther Jung, Christian Freund, Kirsten Falk, Olaf Rötzschke, Ronald Kühne

**Affiliations:** 1 Leibniz-Institute for Molecular Pharmacology (FMP), Berlin, Germany; 2 Max-Delbrück-Center for Molecular Medicine (MDC), Berlin, Germany; 3 Dr. Panjwani Center for Molecular Medicine & Drug Research, International Center for Chemical & Biological Sciences, University of Karachi, Karachi, Pakistan; 4 Department for Disease Biology, Faculty of Life Sciences, Copenhagen University, Copenhagen, Denmark; 5 Eberhard-Karls University, Tübingen, Germany; 6 Singapore Immunology Network (SIgN), Agency for Science, Technology and Research (A*STAR), Singapore, Singapore; University of South Florida College of Medicine, United States of America

## Abstract

MHC class II molecules (MHC II) play a pivotal role in the cell-surface presentation of antigens for surveillance by T cells. Antigen loading takes place inside the cell in endosomal compartments and loss of the peptide ligand rapidly leads to the formation of a non-receptive state of the MHC molecule. Non-receptiveness hinders the efficient loading of new antigens onto the empty MHC II. However, the mechanisms driving the formation of the peptide inaccessible state are not well understood. Here, a combined approach of experimental site-directed mutagenesis and computational modeling is used to reveal structural features underlying “non-receptiveness.” Molecular dynamics simulations of the human MHC II HLA-DR1 suggest a straightening of the α-helix of the β1 domain during the transition from the open to the non-receptive state. The movement is mostly confined to a hinge region conserved in all known MHC molecules. This shift causes a narrowing of the two helices flanking the binding site and results in a closure, which is further stabilized by the formation of a critical hydrogen bond between residues αQ9 and βN82. Mutagenesis experiments confirmed that replacement of either one of the two residues by alanine renders the protein highly susceptible. Notably, loading enhancement was also observed when the mutated MHC II molecules were expressed on the surface of fibroblast cells. Altogether, structural features underlying the non-receptive state of empty HLA-DR1 identified by theoretical means and experiments revealed highly conserved residues critically involved in the receptiveness of MHC II. The atomic details of rearrangements of the peptide-binding groove upon peptide loss provide insight into structure and dynamics of empty MHC II molecules and may foster rational approaches to interfere with non-receptiveness. Manipulation of peptide loading efficiency for improved peptide vaccination strategies could be one of the applications profiting from the structural knowledge provided by this study.

## Introduction

Major histocompatibility complex molecules (MHC) display protein fragments on the cell surface for the surveillance by T cells. While class I MHC molecules present mainly peptides of endogenous origin, the majority of the ligands of class II MHC molecules (MHC II) derives from exogenous proteins. After internalization into endosomal compartments these proteins are proteolytically degraded into shorter fragments and loaded onto MHC II molecules. The peptide/MHC II complexes are then transported to the cell surface where they are displayed to CD4^+^ T cells that detect potential foreign antigens [1].

In the endosome the formation of stable peptide/MHC II complexes is assisted by the chaperone HLA-DM [2]. By interacting directly with the MHC II molecule the chaperone stabilizes a ‘receptive’ conformation that allows rapid ligand exchange. While this promotes binding of ligands with highest affinity, a certain fraction of MHC molecules still looses its ligand causing the appearance of ‘empty’ MHC II molecules on the cell-surface [3]. *In vitro* data show that empty MHC II molecules rapidly ‘inactivate’ by acquiring a ‘non-receptive’ conformation [4]. This non-receptive state is characterized by the inability to bind any free peptides and is presumably based on a closure of the peptide binding cleft [4], [5]. While on the one hand, this may prevent ‘accidental’ loading of these molecules with peptides of the extracellular space, it could also hamper efficient loading of APCs during peptide vaccination.

Despite the fact that both the receptive and the non-receptive states are kinetically well defined, their structures as well as the underlying mechanism of inactivation is not known [6], [7]. Numerous crystal structures have been solved for peptide-loaded MHC II molecules but so far empty MHC II has resisted all crystallization attempts. To gain insight into the structure of the non-receptive MHC II conformer, we carried out molecular dynamics (MD) simulations using a peptide-stripped human MHC II molecule (HLA-DR1). The *in silico* model derived from these calculations revealed a very defined conformation, in which entrance to the peptide-binding site is blocked primarily by a shift of the β1 α-helix. This helix adopts an energetically favored extended conformation leading to a narrowing of the β1- and α1-helix flanking the peptide-binding site. The concomitant groove closure is stabilized by a newly formed H-bond network attaching the conserved helix residue βN82 to residues located on the floor of the binding site. Site-directed mutagenesis experiments of soluble and cell surface MHC II confirmed that the integrity of this ‘lock’ is indeed crucial to maintain the non-receptive state.

## Results

### Molecular Dynamics (MD) simulations of “empty” MHC II molecules

Extended molecular dynamics (MD) simulations of ‘empty’ HLA-DR1 molecules were carried out to identify characteristic features of non-receptive MHC II molecules. ‘Empty’ starting structures were generated from published X-ray crystal structures by removing the peptide from the MHC binding site in silico. In order to avoid computational bias resulting from crystal packaging or from ligand-dependent side chain orientations, coordinates of two different MHC II/ligand complexes were used (PBD entry 1DLH: HLA-DR1/HA306-318 [8] and 1SJE: HLA-DR1/GAG166-181 [9]). To further improve the significance of the calculation, between 2 and 5 independent simulations were performed (Table 1). As reference for the non-receptive structure MD simulations were also carried out with HLA-DR1 molecules with a ligand-stabilized receptive conformation. In previous studies we have shown that the occupation of the anchor pocket P1 by small organic molecules such as AdEtOH (2-(1-adamantyl)ethanol) [10] or short peptide fragments [11] maintains the receptive state of the MHC molecule. Compounds exhibiting this effect had been termed ‘MHC loading enhancer’ (MLE) [10] . For this study the dipeptide Ac-FR-NH_2_, known to act as an MLE on HLA-DR1 molecules, was docked to the P1 pocket to generate model structures representing the empty receptive state.

**Table 1:** Overview of the calculated MD-run ensembles.

name of run	PDB entry of HLA-DR1 complex	number of runs	simulation time (ns)
DR1_1DLH_/HA306-318	1DLH (with HA306-318 peptide)	2	15
‘empty’ DR1_1DLH_	1DLH (without peptide)	5	30
DR1_1SJE_/GAG166-181	1SJE (with GAG166-181 peptide)	2	15
‘empty’ DR1_1SJE_	1SJE (without peptide)	4	30
DR1_1DLH_/Ac-FR-NH_2_	receptive 1DLH (docked *in silico* with Ac-FR-NH_2_ MLE compound)	3	15

**Figure 1:**
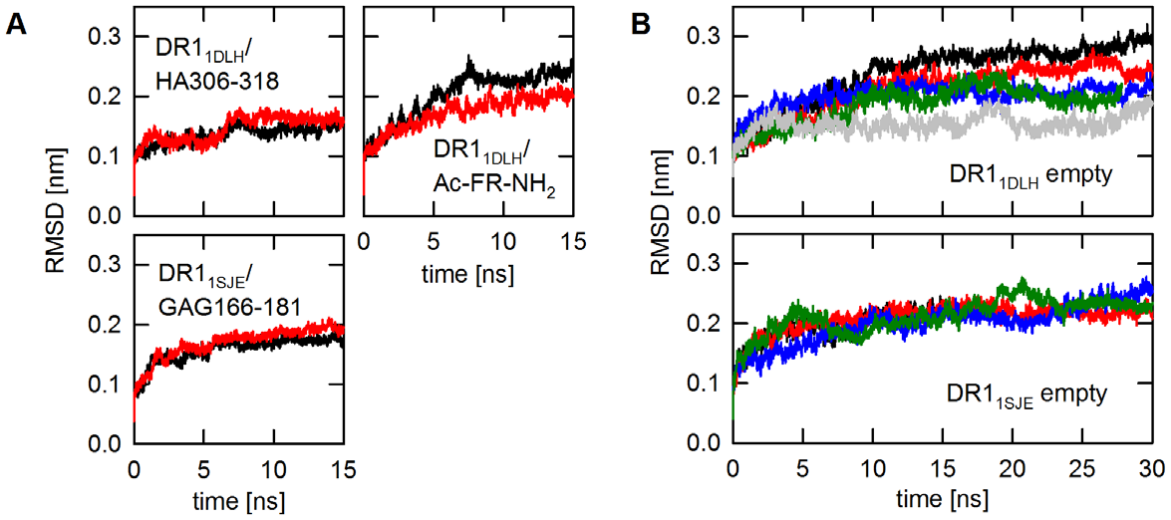
Root mean square deviations (RMSD) of the molecular dynamics (MD) simulations for the individual HLA-DR1 ensembles. RMSD values for the α1/β1-domain are shown for the time course of the simulation. Different colors represent individual runs. **A**, The two peptide-loaded structures DR1_1DLH_/HA306-318 and DR1_1SJE_/GAG166-181 (left panels) and the P1-stabilized dipeptide-loaded model DR1_1DLH_/Ac-FR-NH_2_ were calculated for 15 ns (right panel). **B**, Empty HLA-DR1-models derived from PDB entries 1DLH (DR1_1DLH_ empty) and 1SJE (DR1_1SJE_ empty) were calculated for 30 ns.

The MD simulations covered trajectories up to 30 ns. Based on root mean square deviations (RMSD) this time frame was sufficient for all ensembles to reach conformational stability (Fig. 1). The two peptide-loaded HLA-DR1 ensembles (DR1_1DLH_/HA306-318 and DR1_1SJE_/GAG166-181) as well as the P1-stabilized receptive form (DR1_1DLH_/Ac-FR-NH_2_) reached the equilibrium at ∼5 or ∼10 ns, respectively (Fig. 1A). A substantially slower equilibration was observed for the two sets of empty MHC II molecules (DR1_1DLH_ and DR1_1SJE_); an effect caused by the higher flexibility of the peptide free ensembles (Fig. 1B). To increase the coverage of the conformational space the number of computational runs for the empty forms was raised from 2 to 5.

**Figure 2:**
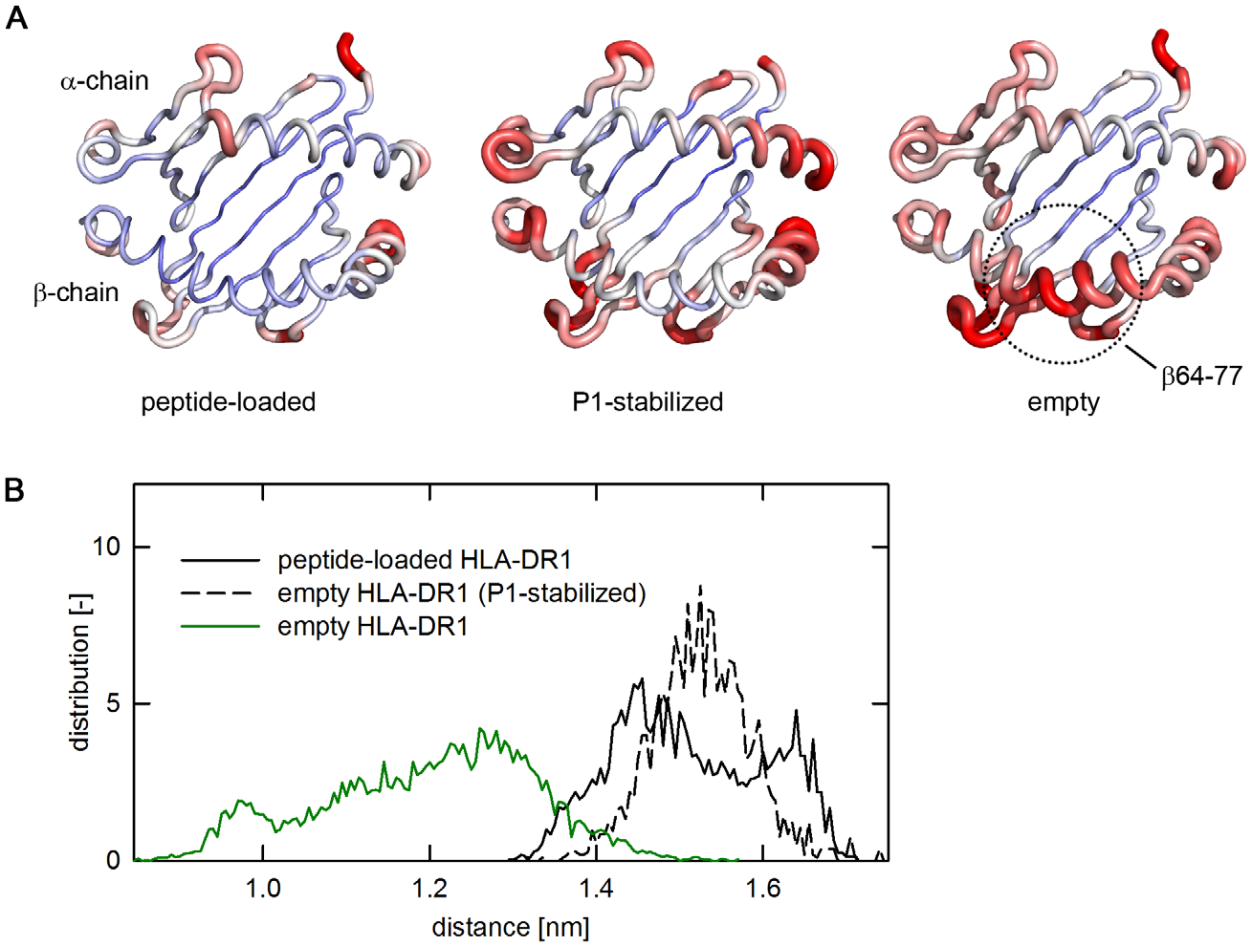
Empty HLA-DR1 shows high mobility in the helices proximal to the P1 pocket. **A**, Color-coded view of the flexibility of the antigen-binding site. The backbone structure of the peptide-binding site (α1/β1-domain) of HLA-DR1 is shown. In this orientation the upper α-helix is formed by the α-chain, the lower α-helix by the β-chain, the N-terminus of the peptide ligand would point to the left side. The chains are colored as heatmaps based on the RMSD of individual Cα-atoms from blue (small RMSD) to red (RMSD≥6 Å). The thickness of the backbone tube varies accordingly (left panel, peptide loaded HLA-DR1 molecule; middle panel, the P1-stabilized receptive form; right panel, the empty form). For the empty form, the hinge region (β64–77) showing the most extensive movement is indicated. **B**, Closure of peptide-binding site by helix segments flanking the P1-pocket. The distance-distribution of the two helices flanking the P1-pocket (α51–61/β73–85) is shown for the average of the indicated simulations. The distribution is shown for the empty HLA-DR1 (green, solid line), the peptide-loaded form (black, solid line), and the P1-stabilized receptive form (black, dashed line).

While the two peptide-loaded structures showed RMSD values of only 0.05 to 0.18 nm for the backbone, the two ‘empty’ HLA-DR1 structures DR1_1DLH_ and DR1_1SJE_ showed a substantially higher flexibility even after 10 ns equilibration time. Here, RMSD values varied between 0.17 and 0.3 nm (Fig. 1B; overlays of frames representing the start- and the equilibrated end-structure of all the ensembles are depicted in supplementary Fig. S1). A heatmap of the individual RMSD values plotted onto the MHC structure revealed the RMSD variations to be mainly caused by movements in the α-helices flanking the peptide-binding site (Fig. 2A). The most striking differences between empty and loaded MHC molecules were observed in the region β64–β77 of the β1 α-helix (compare Fig. 2A, left and right panel). This segment is located proximal to pocket P1 and ranges from a smaller kink in the α-helix close to P1 to a prominent kink, present in all known MHC molecules. While only very limited movements were observed in the peptide loaded form (Fig. 2A, left panel), the P1-stabilzed form showed a slightly increased flexibility (Fig. 2A, middle panel). This, however, was mostly restricted to the helical regions located distal to P1 but no substantial shifts were recorded in the region β64–β77.

Distance measurements between the segments flanking the P1 pocket (α51–α61 and β73–β85) showed that the β64–β77-movement caused a narrowing of the two opposing helix segments which indeed resulted in a closure of the binding site (Fig. 2B and supplementary Fig. S2 for the individual runs). The distribution of observed distances for all simulated frames revealed a main distance of only 1.1–1.3 nm for the empty HLA-DR1 molecule. In contrast, in the peptide-loaded, as well as in the simulation of the receptive P1-stabilized form, the majority of the frames exhibited a significantly larger distance ranging from 1.4–1.6 nm.

### Model of the non-receptive conformation

**Figure 3:**
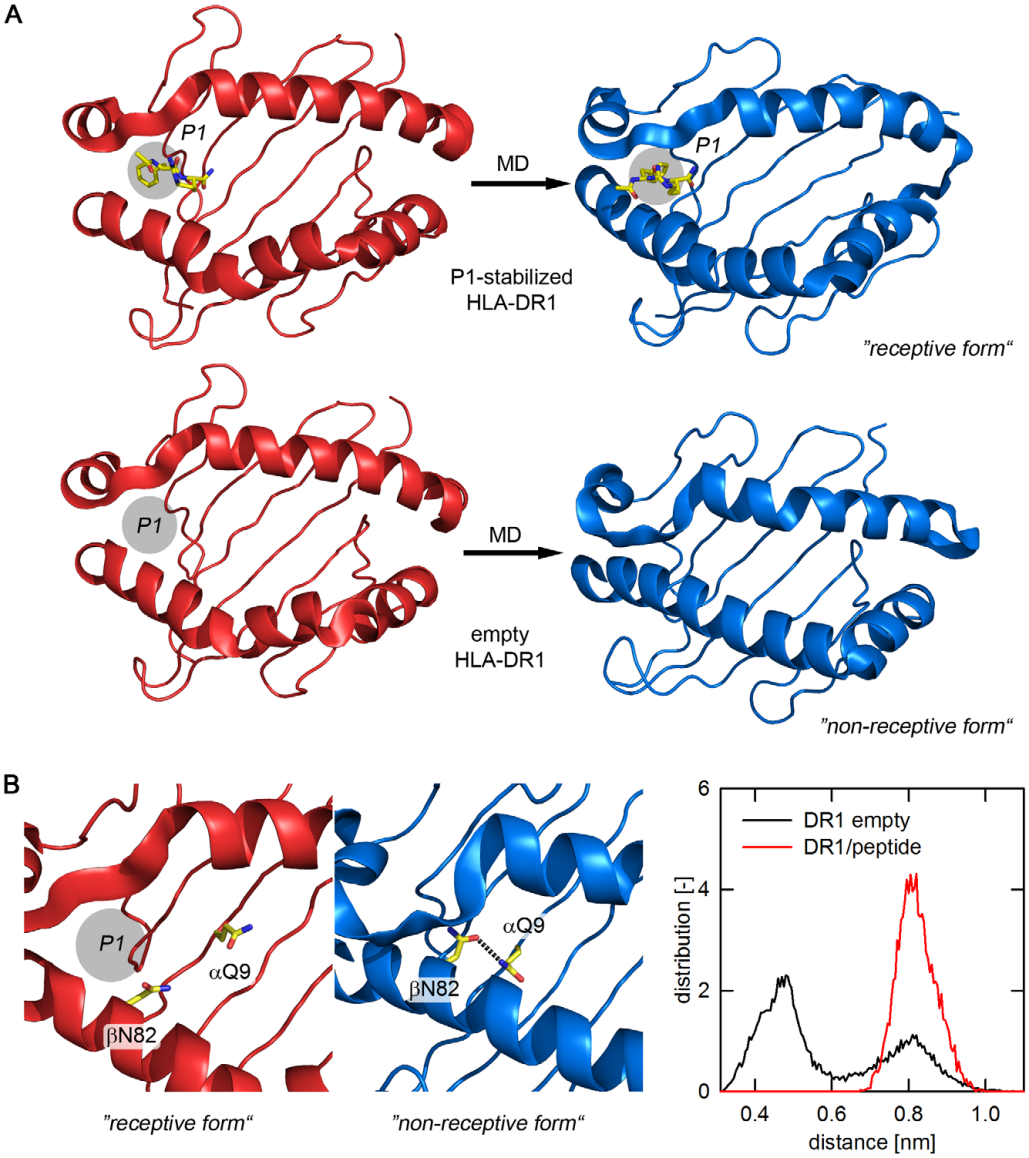
Structural model of the non-receptive conformation. **A**, Overview of structural changes in the antigen-binding site of the P1-stabilized HLA-DR1 (upper panel) and the empty HLA-DR1 (lower panel). The starting structures for the MD simulations are shown in red, the stable end frames of representative runs are shown in blue. The P1-stabilizing dipeptide FR [11], is indicated as yellow stick model. For empty HLA-DR1 the approximate location of the P1-pocket is indicated with a circle. **B**, Stabilization of the closure of the antigen-binding site by the βN82/αQ9 H-bond. The positions of αQ9 and βN82 are shown as yellow sticks. The left panels show close-up views of the postulated binding site structure proximal to the P1-pocket. The region is shown in the receptive (red, left panel) and non-receptive state (blue, right panel). Formation of an H-bond is indicated by a dashed line. Right panel: distribution plot for distances between the centers of the two carboxamides of αQ9 and βN82. Analysis was carried out based on the MD simulations of the peptide-loaded (red line) and empty forms (black line). The distance plot reveals two maxima, with the maximum at the smaller distance (∼4.5 Å) being consistent with the formation of an H-bond.

Graphic models of the MD-based transition to the non-receptive state are depicted in figure 3. While the occupation of P1 by the MLE-dipeptide was sufficient to keep the binding site in an open conformation (Fig. 3A, upper panels), a closure of the binding site was observed in all equilibrated structures of the empty molecule (Fig. 3A, lower panels). Notably however, this closure was not caused by an uncontrolled collapse but rather due to the acquisition of a defined conformational state. The narrowing of the two flanking α-helices, caused mainly by movements in the β64–β77 region, was driven by the acquisition of an energetically favored extended conformation of the β1 α-helix (Fig. 3A, lower panels). This translocation alone is fully sufficient to block peptide entry to the binding site. The process, however, was accompanied by rearrangements of residues inside the binding cleft causing also the previously reported loss of the important P1 anchor pocket [11].

Detailed analysis of the non-receptive frames further suggested that the closed conformation was stabilized by a newly formed H-bond network. An H-bond was formed between βN82 of the β1 α-helix and residue αQ9 located proximal to P1 at the floor of the peptide-binding site (Fig. 3B, left panels). In the peptide-loaded complex both βN82 and αQ9 are involved in an intermolecular H-bond network, attaching the backbone of the peptide ligand to the binding site. After removal of the ligand both residues are exposed to the solvent but quickly form an H-bond stabilizing the extended conformation of the β1 helix segment that closes the binding cleft. The distance-distribution plot for the side chain head groups of βN82 and αQ9 (Fig. 3B, right panel) indicates that in the simulations of the empty state the distance between the two residues is consistent with an H-bond formation, suggesting that it indeed functions as a “lock” for the closed conformation.

#### Experimental evidence for the conformational transitions

**Figure 4:**
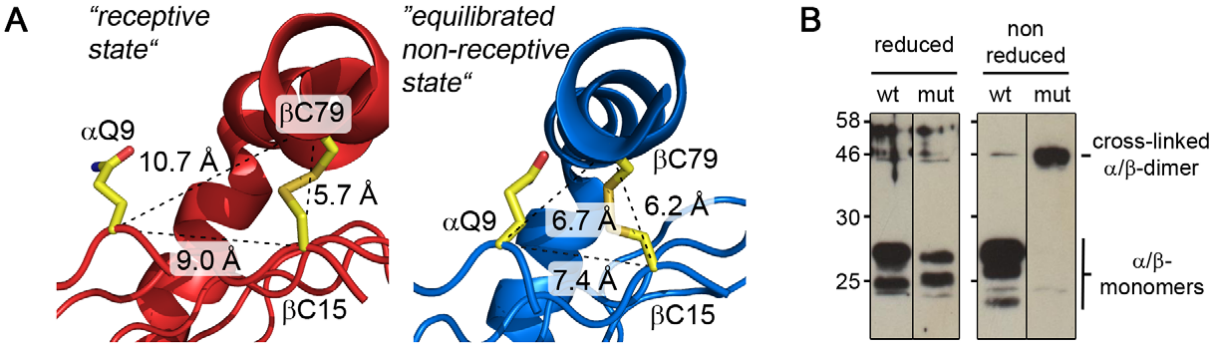
Validation of the helical mobility by experimental introduction of an intermolecular disulfide bond. **A**, Postulated positions of residues αQ9 and βC79 in the receptive and the non-receptive state. The backbone of the α- and the β-chain is shown in red (receptive) and blue (non-receptive), respectively. The perspective is chosen to point into the binding cleft near the P1 pocket, with the β1 α-helix forming the right boundary. Side chains are shown for residues αQ9 and βC79 as well as for βC15, naturally forming an intrachain disulfide bridge with βC79. The Cα-distances are indicated. The receptive state is shown on the left side and the non-receptive state, characterized by a shift of the α-helix towards the center of the cleft, on the right side. **B**, SDS-PAGE analysis of the αQ9C/βC15S mutant. Lysates of cells expressing either HLA-DR1 (wt) or the double mutant αQ9C/βC15S (mut) were separated by SDS-PAGE under reducing (left panel) and non-reducing conditions (right panel). The detection of bands representing either the single or the cross-linked α- and β-chains of HLA-DR1 was done by Western blot using polyclonal HLA-DR-specific serum. Apparent molecular weight and positions of molecular weight markers (in kDa) are indicated.

To provide experimental evidence in support of the postulated shift of the β1 α-helix segment, the non-receptive MHC II structure was inspected for residues suitable for probing the transition in mutagenesis experiments. αQ9 is located close to P1 on the floor of the peptide-binding site (Fig. 4A). In the peptide-loaded as well as in the P1-stabilized receptive state the Cα-atom of αQ9 is more than 10 Å apart from the Cα-atom of βC79, as suggested by MD simulations. This distance would be reduced to about 6.6 Å in the postulated non-receptive conformation, sufficiently close to allow for the formation of an interdomain disulfide bridge [12]. To determine if this bridge can actually be formed, αQ9 was replaced by cysteine. To free βC79 for this interaction its natural disulfide partner βC15 was replaced by serine. After mutagenesis the protein was expressed in baculovirus-infected insect cells and the formation of the interchain disulfide bridge was analyzed in a Western blot by using HLA-DRαβ-specific antibodies after SDS-PAGE (Fig. 4B).

As expected, two bands representing the individual α- and β-monomers for both mutant and wt forms were detected when separated under reducing conditions (Fig. 4B). Analogous bands were also produced by the wild type under non-reducing conditions. However, while dissociation of the two chains was still observed under oxidizing conditions, a single band with a MW_app_ of ∼50 kDa, approximately double the size of the monomeric α/β-chains (28 and 25 kDa, respectively) was detected under non-reducing conditions for the mutated protein species. Separate staining against the α- and the β-chain verified the presence of both chains in the higher molecular weight band, confirming that the complex is indeed representing a disulfide-bridged α/β-heterodimer (data not shown).

#### Experimental evidence for the βN82-lock

**Figure 5:**
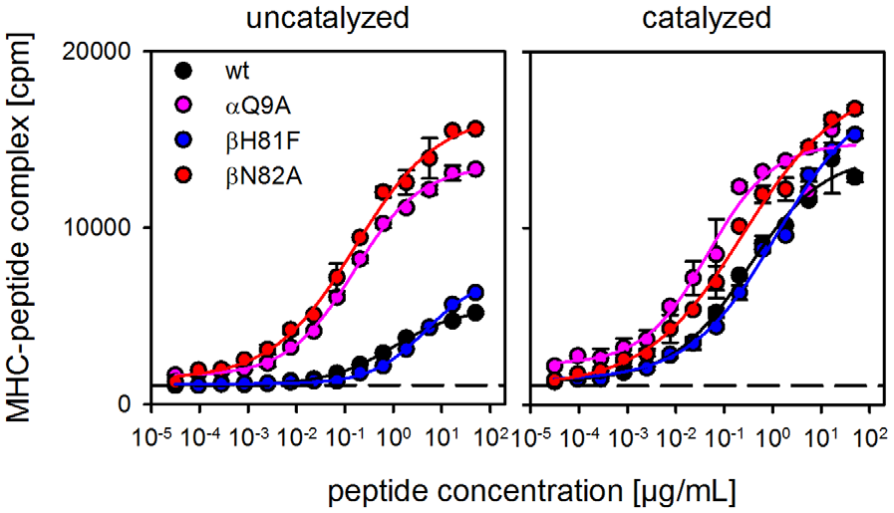
Influence of αQ9 and βN82 on the receptiveness of soluble HLA-DR1. ELISA-based antigen loading assays were carried out with soluble forms of wt HLA-DR1 (black) and mutants αQ9A (magenta), βN82A (red), and βH81F (blue). MHC molecules derived from insect cells were loaded with biotinylated peptide HA306-318. Loading was carried out in the absence (left panel) or presence (right panel) of the ‘MHC-loading enhancer’ (MLE) compound AdEtOH [10]. The dashed line indicates the background in the absence of ligand.

To provide experimental evidence for the importance of the βN82-lock for the stability of the non-receptive state a number of mutations was introduced into soluble HLA-DR1. The specific location of all residues mutated in this study is depicted in supplementary Fig. S3. The side chain positions are shown for both the receptive and the non-receptive conformation suggested by the MD simulation. Receptiveness of the mutated molecules was tested in an ELISA-based peptide-loading assay using a biotinylated form of the peptide antigen HA306-318 (bHA) (Fig. 5).

In the first series of experiments the two residues actually forming the H-bond, βN82 and αQ9, were replaced by alanine. In addition, βH81 was mutated to phenylalanine, an isosteric form of histidine lacking the H-bond forming ability. Previous studies have implicated the H-bond forming capacity of this residue to be involved both in HLA-DM-mediated ligand release [13] as well as in the stabilization of the closed MHC II conformation [14]. In our model this residue is exposed to solvent and should therefore not contribute to the stabilization of the non-receptive state.

Empty MHC II exists in two forms, the peptide-receptive and the non-receptive state. The transition from the non-receptive to the receptive state is rather slow so that the equilibrium between both forms lies largely on the non-receptive side [4]. Thus, empty MHC II is apparently “trapped” in the thermodynamically stable non-receptive state and it is therefore not surprising that wt HLA-DR1 showed rather inefficient peptide binding (Fig. 5, left panel). The same, however, applied also to the βH81F mutant. In contrast to a previous report [13] the absence of the H-bond provided by βH81 had virtually no effect on the loading rate. Contrary to this, however, αQ9A and βN82A clearly exhibited improved peptide-loading capacity. Under the given experimental conditions, comparable peptide loading was achieved at 10^2^–10^3^ times lower peptide concentrations. The receptiveness of wt and βH81F could be restored by the addition of P1-targeting MLE compounds. AdEtOH [10] almost completely eliminated the differences in peptide loading, showing that in principle all constructs were able to acquire the receptive state (Fig. 5, right panel).

**Figure 6:**
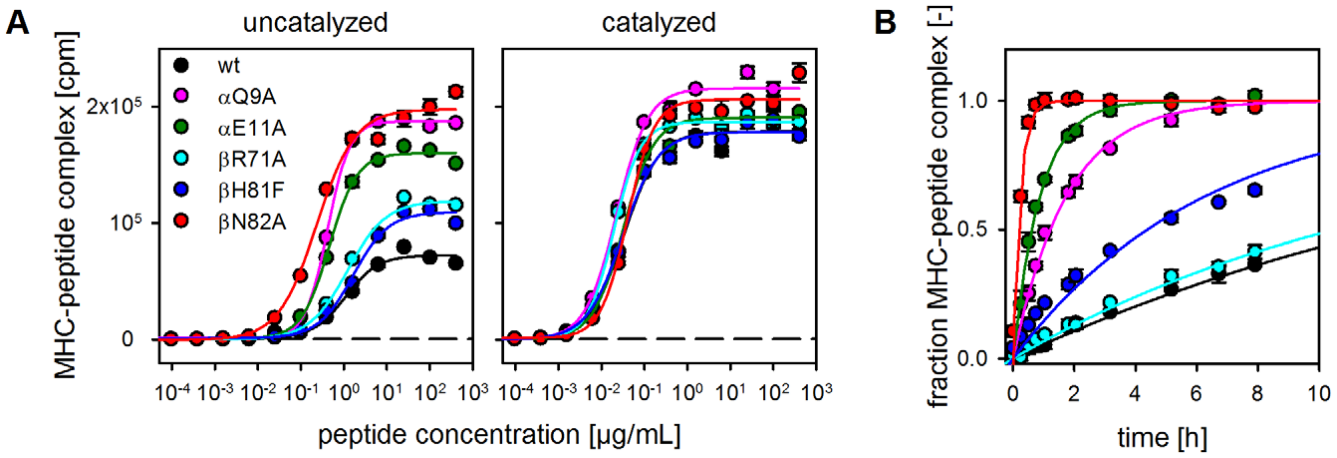
Analysis of αQ9/βN82-interaction with DR1 produced in *E.coli*. **A**, ELISA-based peptide loading assay. Empty, soluble HLA-DR1 molecules refolded from material produced in *E.coli* were used in an ELISA-based antigen loading experiment as described in Fig. 5. wt HLA-DR1 (black), αQ9A (magenta), αE11A (green), βR71A (light blue), βH81F (dark blue) and βN82A (red) were loaded with biotinylated HA306-318. Loading was carried out in the absence (left panel) or presence (right panel) of the MLE-compound AdEtOH. **B**, Kinetic analysis of antigen-loading monitored by fluorescence polarization. wt and mutant forms of HLA-DR1 were loaded with a fluorescence-labeled variant of HA306-318. Loading was monitored in real time by measuring the change in fluorescence polarization. The data normalized to maximum loading were fitted to a single exponential binding function [42].

As mentioned before, the two residues βN82 and αQ9 involved in the locking mechanism are also known to participate in direct ligand interactions. This applies particularly to βN82, which forms two H-bonds with the peptide backbone [8]. MHC II preparations from baculovirus-infected cells as used for the above experiment can contain endogenous ligands. To exclude that the observed increase in loading efficiency of βN82- and αQ9-mutants is only due to the accelerated release of prebound ligands, the experiments were repeated with MHC II molecules produced in *E.coli* (Fig. 6). Here, the α- and β-chain of the MHC molecule are expressed independently followed by purification and refolding of the empty MHC II molecule. *E.coli* HLA-DR1 is therefore almost guaranteed to be devoid of any co-purified peptide ligands [15]. Moreover, to further consolidate our model by separating it from previously published MD simulations two additional mutations were included (βR71A and αE11A). Painter et al. [14] suggested that non-receptiveness is largely due to the P1 blockage by an unfolded α-helical segment, an effect not observed in our simulation (see supplementary Fig. S3). βR71 has been suggested as one of the key-residues stabilizing this blockage but does not play a role in our model. αE11, in turn, has not been implicated in the previous study but is assumed to have a strong indirect influence on the stability of the postulated βN82-lock. While it does not directly participate in the H-bond formation it closely interacts with αD66 as well as αQ9 and is therefore expected to have a profound impact on the electrostatics in this region [16].

When probing the receptiveness of empty *E.coli*-derived MHC II molecules a similar picture emerged as it was observed with material derived from baculovirus-infected cells (compare Fig. 5). In ELISA-based assays the most efficient peptide loading was detected with βN82A followed by αQ9A and αE11A (Fig. 6A). Only a small effect was observed with βR71A and βH81F. As shown before for the baculovirus-derived material the addition of AdEtOH leveled out the differences between the constructs by increasing the receptiveness of wt HLA-DR1, βH81F, and βR71A.

Similar differences in the receptiveness of the mutant forms were also observed when the loading reaction was monitored by fluorescence polarization (FP). This approach allows a precise kinetic analysis since peptide loading can be recorded in real-time (Fig. 6B, Table 2). Half-maximal loading (t_1/2_) required about 12 h for the HLA-DR1 wt form, while less than 0.2 h was needed for the βN82A mutant. αE11A and αQ9A also displayed significantly decreased t_1/2_ (0.7 and 1.3 h, respectively) while βR71A and βH81F required 15.5 and 5.5 h for half-maximal loading, respectively. Thus, while for the latter two only marginal increases were observed it translates into a 60fold faster loading reaction for the βN82A mutant.

**Table 2:** Loading rates and half-maximal loading time of HLA-DR1 variants loaded with HA306-318 as determined by fluorescence polarization.

HLA-DR1	rate [1/h]	t_1/2_ [h]
wt	0.06±0.01	12.2±3.0
αQ9A	0.54±0.05	1.3±0.1
αE11A	1.05±0.1	0.7±0.1
βR71A	0.07±0.01	15.5±1.6
βH81F	0.13±0.02	5.5±0.9
βN82A	3.83±0.66	0.2±0.03

The mutations also affected the thermal stability of the MHC. In a thermal shift assay (TSA) the integrity of the complex in response to a raise in temperature was probed with SyproOrange. The melting point of empty wt HLA-DR1 was determined as ∼66°C. Removal of any of the residues assumed to be involved in the βN82-lock led to a steep drop of thermal stability of more than 13 K (βN82A: 51°C, αQ9A: 52°C and αE11A: 52°C). The loss of βR71and βH81, in contrast, resulted in a much smaller reduction in thermal stability (βR71A, βH81A: 63°C). A similar result was obtained, when the stability was analyzed by dynamic light scattering (DLS). The elimination of any of the three residues involved in the βN82-lock resulted in a significantly lowered melting point as well, while only a marginal change was observed for βH81F (Table 3).

**Table 3:** Melting points of empty HLA-DR1 variants as determined by thermal shift assay (TSA) and dynamic light scattering (DLS).

HLA-DR1	T_m_ [°C] by TSA	T_m_ [°C] by DLS
wt	66.4±0.5	71.5
αQ9A	52.2±0.3	55
αE11A	51.8±0.2	55.3
βR71A	62.9±0.8	n.d.*
βH81F	62.5±0.4	65.5
βN82A	51.3±0.1	51.1

*) not determined.

#### The βN82-lock controls receptiveness on cell surface MHC II molecules

To this point, all of our studies had been performed with soluble HLA-DR1 molecules lacking intracellular and transmembrane regions. To determine whether the postulated locking mechanism also controls receptiveness on the cell surface HLA-DR1 wt and mutants were expressed as full length proteins in murine fibroblast cells. In a FACS-based *in vitro* loading assay [10] the cells were incubated with biotinylated HA306-318 peptide and the amount of peptide loaded onto the cells was determined by flowcytometry after staining with fluorescence-labeled streptavidin.

**Figure 7:**
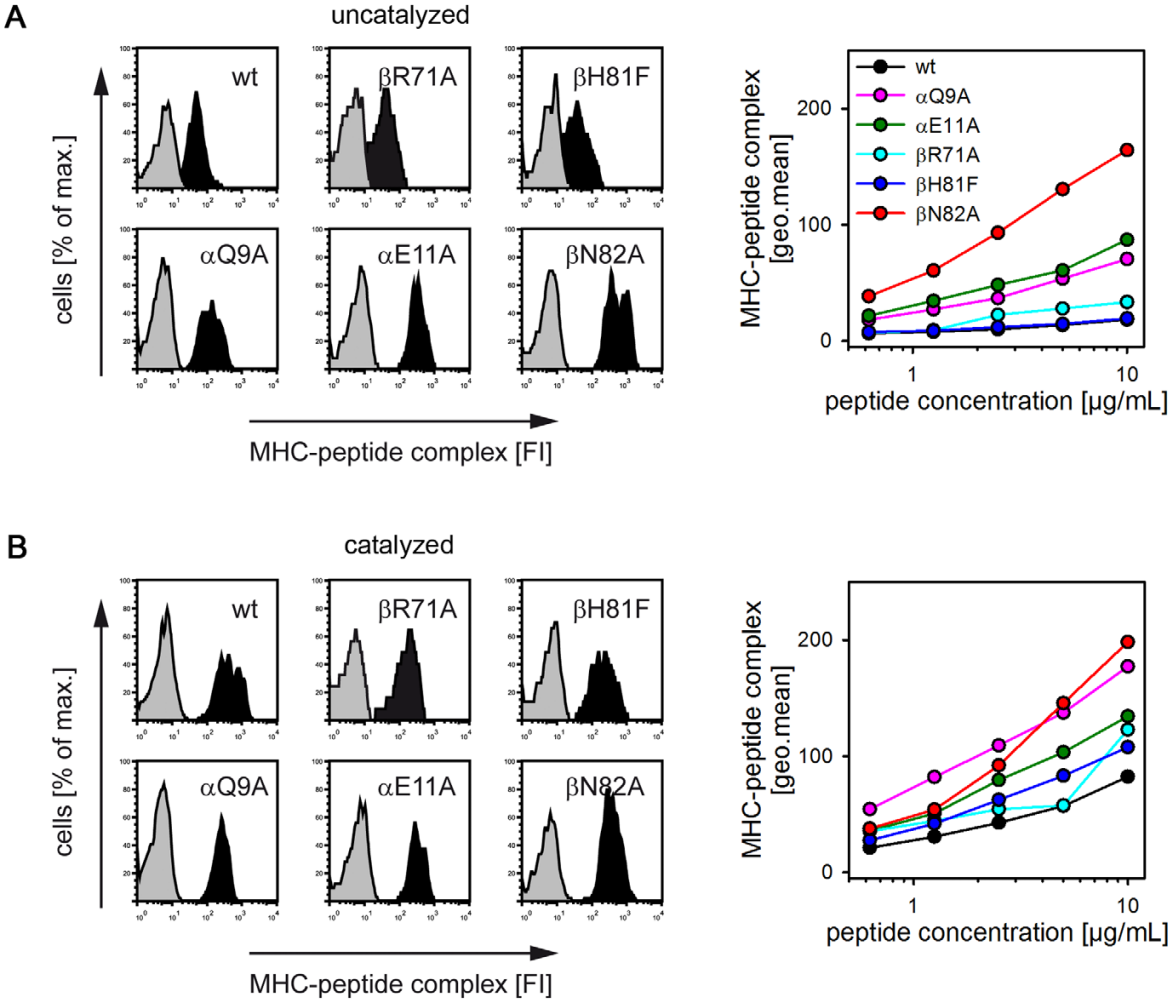
Influence of the putative βN82-lock on the receptiveness of cell surface MHC II. **A**, Surface loading of HLA-DR1-expressing fibroblast cells. Full-length forms of wt and mutant HLA-DR1 were expressed in L929 fibroblast cells. In an *in vitro* loading assay [10], cells were incubated for 4 h with biotin-labeled HA306-318. Cell-surface loading of HLA-DR1 with peptide was analyzed by FACS after staining with APC-streptavidin. Representative histogram plots (right panels) are shown depicting the fluorescence recorded in the absence (grey) or presence of the biotinylated HA306-318 peptide (black). Dose response curves for titrated amounts of the peptide are shown in the right panels for wt HLA-DR1 (black), αQ9A (magenta), αE11A (green), βR71A (light blue), βH81F (dark blue), and βN82A (red). **B**, Cell surface loading in the presence of MLE. The same experiments as described in Fig. 6A were carried out in presence of the MLE compound AdEtOH.

The histogram of the streptavidin staining revealed that only low amounts of peptide can be loaded onto cells expressing the wt MHC II (Fig. 7A, left panels). Substantially more peptide was detected on cells expressing βN82A, αQ9A and αE11A, while almost no increase was observed for βH81F and βR71A. The differences are even more evident when comparing the loading reactions for the different MHC variants in a dose-dependent way (Fig. 7A, right panel). Mutations disrupting the proposed H-bond network resulted in a 9fold increase for βN82A and a 4–5fold increase for αQ9A and αE11A. In contrast βH81F and βR71A-mutations did not significantly affect peptide binding. As shown before for soluble MHC II, functionality of all mutants was verified by addition of AdEtOH (Fig. 7B). Thus, the βN82-lock seems to be functional also on the cell-bound form of MHC II and disrupting this lock leads to an increased loading efficiency of antigens. Subsequently, the higher peptide/MHC density on the cell surface is likely to enhance the T cell response. This particularly applies at lower antigen concentrations.

## Discussion

The study strongly suggests that the receptive as well as the non-receptive state of empty class II MHC molecules are based on conformation with very distinct structural features. The *in silico* analysis of a molecular dynamics simulation of empty MHC II indicates that the closure of the binding site is likely being caused by defined structural transitions, namely the extension of the β1 α-helix at a prominent kink near residues β70–75. The narrowing of the two helices flanking the peptide-binding region results in a closure of the site, a conformational shift, stabilized by the formation of an H-bond lock involving the conserved helix residue βN82. While the models derived from MD simulations identified single residues governing the inactivation of MHC II, we could also provide experimental evidence that the mutation of key residues identified by the *in silico* approach strongly affects the acquisition of the non-receptive state.

Kinetically differentiable forms of empty MHC II are well known [4], [5], [16] and structural alterations in the conformation have been made responsible for the apparent differences between receptive and non-receptive forms [6], [7]. However, despite several attempts to reveal the conformational changes [6], [17], [18] the precise nature of the underlying structural change has remained elusive. As direct structural data is lacking, molecular dynamics simulation has recently been applied as an alternative to gain some insight into the structural dynamics of empty MHC II [11], [14], [19]. Several models have been proposed. Painter et al. suggested that the non-receptive state is characterized by partial unfolding of the α1-helix [14]. This unfolded segment occupies the binding site, stabilized by H-bond interactions with a set of residues (αQ9, βR71, βH81, and βN82), in a fashion closely resembling peptide binding. In initial MD simulations partial unfolding was also observed by Yaneva et al. [19]. This, however, could be corrected by computational adjustment of the protonation state of the starting structure. Similarly, our own simulations did not produce any evidence for the postulated α-helix unfolding. The integrity of secondary structure elements was maintained during the simulation. Moreover, mutagenesis of two residues involved in the stabilization of the unfolded complex (βH81 and βR71) did not reveal any influence on the receptiveness of the empty MHC molecule (Fig. 6). This applies in particular to βH81 which had even been suggested by others to be the key-factor in controlling the ligand exchange rate [13]. In addition to inconsistence with the data presented in this study, two other recent studies also indicate that βH81 is only of minor importance for the peptide-exchange [20], [21].

Our data instead support a model in which the transition from the receptive to the non-receptive state is more subtle and characterized only by defined shifts of conserved secondary structure elements. Central to this is the shift of the β1-helix resulting in a closure of the binding cleft. This shift is accompanied by a loss of the P1 pocket, mainly as a consequence of a rearrangement of other residues in the binding site. The movement is largely confined to the β64–β77 segment leading from a bend conformation to the acquisition of an energetically favored extended conformation. Notably, this straightening of the helix is not observed when the P1 pocket is filled by a short dipeptide. The open conformation reported here and in a previous simulation [11] was also observed by Yaneva et al. in an MD simulation of HLA-DR3 loaded with a dipeptidic CLIP fragment [19].

As an important structural feature the MD simulation suggested an H-bond lock in which the conserved helix residue βN82 forms a bridge with αQ9. The H-bond seems to stabilize the closed conformation by fixing the extended β1 α-helix to the floor of the empty binding site. Experimental evidence provided by the mutational analysis of HLA-DR1 confirmed that these residues play indeed a significant role for the stability of the non-receptive state. Ablation of this interaction led to empty MHC molecules with a highly increased peptide-loading efficiency. This applied both to truncated soluble molecules as well as to membrane-bound, full length MHC. A similar enhancement was also observed for αE11, a residue located proximal to αQ9 interacting with αD66 of the α1 α-helix. The residue is expected to modulate the strength of the αQ9/βN82 bridge by electrostatic interaction. Interestingly, one report already discussed the potential influence of the αE11/D66-cluster in I-E^k^ as being responsible for a conformational change from a peptide-exchange-susceptible to a stable, exchange-insusceptible peptide/MHC-conformer [22].

The impact of single MHC II residues on the overall stability of peptide/MHC complexes is well known. Respective interactions were demonstrated for the murine MHC II molecules I-A^d^ [23], [24], [25] and I-E^k^ [26] as well as for human HLA-DR1 [13], [27]. Also in these studies the effect has been attributed to the particular contributions to a conserved hydrogen network that particularly applies to βN82. The mutation of βN82 to serine has a profound influence on peptide-MHC stability as was shown for I-E^d^ and I-A^d^ [28], [29] and the transport of nascent MHC to the cell surface was also affected by this mutation [30]. Central to these previous studies was the H-bond formed between βN82 and the peptide ligand. The data presented here, however suggest that internal H-bonds formed by βN82 and residues on the floor of the binding site may play an equally crucial role in maintaining conformational stability of the empty molecule. Utilizing ‘truly’ empty MHC II derived from *E.coli* we were able to show that interference with the proposed lock significantly reduced the thermal stability of the empty MHC II. Moreover, the empty MHC II from *E.coli* preparations also enabled us to analyze the peptide loading of empty MHC II devoid of any pre-bound ligands. Clearly, with these preparations the high impact of the βN82 lock on the loading efficiency of MHC II could be reproduced. Thus, the H-bond donor function of βN82 seems to be crucial for both fixing the ligand on the MHC molecule and closing the empty binding cleft.

Importantly, the proposed locking mechanism was also detected on membrane-bound MHC II. Loading rate and efficiency were affected by mutations in the same way as for the soluble MHC II. From an immunological point of view the transition from a receptive to a non-receptive state could function as a safeguard mechanism that prevents uncontrolled binding of antigens to empty MHC. Empty, peptide-receptive MHC on the cell surface of an antigen-presenting cell (APC) could bind any suitable antigen from the extracellular milieu. In fact, capture of extracellular antigens by immature dendritic cells has been suggested to be an alternative pathway with particular relevance for tolerance induction [3], [31], [32]. However, as it bypasses defined uptake mechanisms, such as Fc-receptor- or scavenger receptor-mediated antigen uptake it might also lead to the induction of unwanted inflammatory responses. Notably, the group of Unanue showed that the route of antigen presentation can even allow to address different types of T cells. Type A T cells responded to antigens when they were delivered to the MHC molecule after uptake via the endosomal pathway, while Type B T cells responded only to peptides directly loaded onto the MHC [33]. The reason for this “peptide isomerism” is still unknown. It may have conformational causes but might also be due to different orientations of the ligand [33], [34]. Recently they could show that in the Non-Obese Diabetic (NOD)-mouse model both T cell types can be found in diabetogenic mice. Moreover, type B T cells were able to induce diabetes upon adoptive transfer [35].

In summary, we could define discrete conformational states corresponding to the well described kinetic states of empty MHC II. While this model describes ‘non-receptiveness’ in a conclusive way, it may not reflect the end-point of conformational change. However, with βN82, αQ9 and αE11 we identified a set of residues close to the P1 pocket that greatly affects the stability of the non-receptive state presumably by locking the empty MHC molecule by an internal H-bond network. While the acquisition of this non-receptive state may serve as natural safeguard mechanism to prevent autoimmune reactions, in the context of peptide vaccinations it can strongly abrogate effective antigen loading. Understanding the structural basis of the transition may therefore not only provide insight on dynamics and function of MHC-conformers, it may also provide new molecular targets to improve the efficiency of immune interventions.

## Materials and Methods

### Molecular Dynamics simulations

For each ensemble used in the simulation (the empty MHC structure, the MHC-antigen complex, and the Ac-FR-NH_2_ in complex with the MHC) a 15 or 30 ns molecular dynamics simulation was performed using the GROMACS software package with the GROMOS force field (ffG43a1) [36]. The proteins were placed into a triclinic box which is 39% smaller than a rectangular system. The simulations were done under physiological conditions. For this purpose the structures of the proteins and of the ligand complexes were calculated in a 0.9% NaCl (e.g. ∼12700 Spc H_2_O molecules, 70 Na^+^- and 60 Cl^−^-ions, respectively) solution at 310 K and periodic boundary conditions for the box. Short range non-bonded interactions were calculated with the cut-off Lennard-Jones potential up to a distance of 1.2 nm between the interacting atoms. For long range electrostatic interactions the particle mesh Ewald (PME) option was used with a grid spacing of 0.12 nm. The bond length of a heavy atom to a hydrogen atom in protein or water was constrained using the LINCS and SETTLE algorithm, respectively. Each simulation ensemble was energy minimized in a two step minimization strategy. In a first step steepest descent and in a second a conjugate gradient minimizing routine was used. After equilibration over a period of 500 ps using a positional restraint of 1000 kJmol^−1^ nm^−2^ on the backbone of the protein and, if present, on the ligand, a free simulation was performed for a period of 15 ns for ligand complexes and of 30 ns for the empty MHC. Temperature and pressure (1 bar) were controlled using the Berendsen coupling with a relaxation time of 0.1 ps for temperature and 1 ps for pressure. During the course of the simulation, a frame was stored every 5 ps. Visualization of trajectories and arrangement of the figures were realized using VMD [37] and PyMOL (DeLano Scientific, California, USA).

### Generation of MHC molecules

Soluble HLA-DR1 was expressed in a baculovirus-based system or by refolding from *E.coli*. The insect cell derived MHC was expressed essentially as described before [11]. Briefly, for the insect cell derived protein DNA coding for the extracellular domains of DRA*0101 and DRB1*0101 was cloned into the transfer vector pFastbac 1 (Invitrogen). Leucine zipper domains were added to the C-termini of the α- and β-chain, as described [38]. Site-directed mutagenesis of HLA-DR1 was carried out using the QuikChange site-directed mutagenesis kit (Stratagene). Recombinant viruses were generated in *S.frugiperda* cells (Sf21). For expression of proteins, cells were co-infected with viruses for the α- and β-chain. Soluble HLA-DR1 was purified from the culture supernatant by affinity chromatography with the HLA-DR specific antibody LB3.1 as described [39].

Soluble HLA-DR1 from *E.coli* was refolded from inclusion bodies as described previously [40] in a method adapted from Stern and colleagues [15]. The ectodomains of the α- and β-chain were expressed separately as inclusion bodies. After purification of the single chains, both denatured chains were combined and refolded together by dilution. Correctly refolded MHC was further purified as described above.

### Western blot analysis of cross-linked HLA-DR1

For cross-linking experiments HLA-DR1 variants without additional leucine zippers were expressed in baculovirus-infected Sf21 cells. Cells were harvested 72 h postinfection and lysed in a buffer containing 50 mM Tris, pH 8.0, 150 mM NaCl, 1% NP40, and 20 mM NEM. For reduction of samples 100 mM dithiothreitol was added. 1 µg total protein per lane was separated on a 12% SDS-PAGE and subsequently transferred to a polyvinyldifluoride membrane (Immobilon P, Millipore, USA). MHC was detected with a rabbit serum raised against the HLA-DRα- and β-chain as described [41].

### Peptide binding to soluble HLA-DR1

The peptide-binding ability of the different HLA-DR1 variants was evaluated by an ELISA-based assay [10]. Briefly, 100 nM soluble HLA-DR1 from *E.coli* or insect cells were incubated with increasing amounts of biotinylated HA306-318 for 30 min resp. 2 h at 37°C. Afterwards the amount of peptide-loaded MHC was assessed by a DELFIA-based ELISA with the HLA-DR-specific capture antibody L243 and Eu^3+^-labeled streptavidin. The plates were read in a Victor 3V reader (Perkin Elmer). Curves were fitted to a four-parameter logistic function with the SigmaPlot11 (Systat Software Inc.) software. Loading rates of the empty HLA-DR1 variants were determined with a fluorescence polarization assay. In a total reaction volume of 40 µL 1 µM HLA-DR1 was incubated with 100 nM FITC-labeled HA306-318 in a non-binding black microplate (Corning). Measurements were conducted in a Victor 3V reader at 37°C. In between measurements the plate was kept in the dark at 37°C. Data points were fitted to a monomodal association model [FP = FP_max_(1-e^−kt^)] [42], where k is the observed rate constant at the given concentration.

### Peptide binding to full length cell-surface HLA-DR1

Murine fibroblast cells L929 were transfected with two pcDNA3.1 vectors containing the full length HLA-DR1 α- resp. β-chain coding sequences for transient expression of the MHC. The loading experiments were carried out 4 days after transfection. Therefore, 10^5^ cells/well were incubated with increasing amounts of biotinylated HA306-318 (4 h, 37°C, DMEM, 5% FCS). For FACS-analysis cells were stained with streptavidin-PE and analyzed on a FACScalibur instrument (BD Biosciences) as described [10]. Dead cells were excluded by propidium iodide staining. To compensate for the variability in cell-surface expression levels of different transfections cells expressing the different HLA-DR1 variants were gated on a uniform MHC II surface expression level by staining with L243-FITC.

### Thermal stability

For determining the thermal stability of the HLA-DR1 variants a thermofluor assay was used [43]. In the fluorescence-based assay 24 µl of a 0.2–0.4 mg/mL gel filtrated sample were transferred to a microtiter plate (ABGene), mixed with 1 µL 125× SyproOrange (Molecular Probes, Invitrogen) and measured in an iCycler iQ Real Time Detection System (Bio-Rad), using steps of 1°C from 20°C to 99°C. This dye responds to altered chemical environments by a shift in its fluorescence spectrum. Fluorescence intensity curves were plotted versus temperature and midpoints of the protein unfolding transition (T_m_) were determined by fitting a sigmoidal curve to the data. The transition points of the fluorescence intensity over time were defined as T_m_. T_m_-values are the mean values from at least two independent experiments conducted in duplicate. Dynamic light scattering measurements were carried out in the Zeta Nanosizer ZS (Malvern, UK). 50 µL of freshly gel filtrated sample at 1 µM were slowly heated from 25 to 91°C (by three degrees steps from 20 to 46 and two-degrees steps to 90°C). After 10 min equilibration time the samples were measured five times for one minute and the values were averaged. According to manufacturer's recommendation the melting point of the sample was determined by plotting the particle size of the main fraction against the temperature and defined as the point of significant increase with irreversible particle sizes after recooling of the sample to 25°C

## Supporting Information

Figure S1 **Antigen-binding site is closing in the absence of P1-occupation.** The structure of the antigen-binding site of HLA-DR1 in representative frames of the MD simulations is shown. The starting structure is in red, the equilibrated end structure in blue. **A**, Comparison of peptide-loaded (left) and peptide-free (right) simulations of both crystal structures used in this study (1DLH upper panel, 1SJE, lower panel). **B**, Occupation of P1-pocket by the dipeptide leads to stabilization of the whole antigen-binding site during MD.
(TIF)

Figure S2 **Distance of the helical regions flanking the P1-Pocket.** The distance distribution for each individual MD simulation is shown. The distance was measured between the centers of mass of the α-helical regions flanking the P1-pocket (α51–61 and β73–85). The different starting structures are indicated in the graphs, the individual runs are shown in different colors. The distance distribution for the replicated runs is comparable. Only in one out of nine runs with empty HLA-DR1 a very narrow distance was reached (DR1_1DLH_empty, black line, upper left panel). Visual inspection of the corresponding MD simulation reveals in this case a drastic inwards motion of the β-chain α-helix resulting in a collapsed antigen-binding site. The significance of this singular event, however, is not clear.
(TIF)

Figure S3 **Position of residues used for introduction of point mutations to probe the putative locking mechanism.** The amino acids are shown as yellow sticks. Left, open binding site at the beginning of the MD simulations. Right, closed binding site of the equilibrated closed structure of empty MHC II. αQ9 and βN82 are forming an H-bond (black dashed line). Residues βR71 and βH81 are apparently not involved in the stabilization of the non-receptive structure.
(TIF)
