# Supplementary figures and images for: Characterization of Structural Features Controlling the Receptiveness of Empty Class II MHC Molecules
Source: PLoS One. 2011 Apr 14;6(4):e18662. doi: 10.1371/journal.pone.0018662 (PMC3077389; doi:10.1371/journal.pone.0018662)

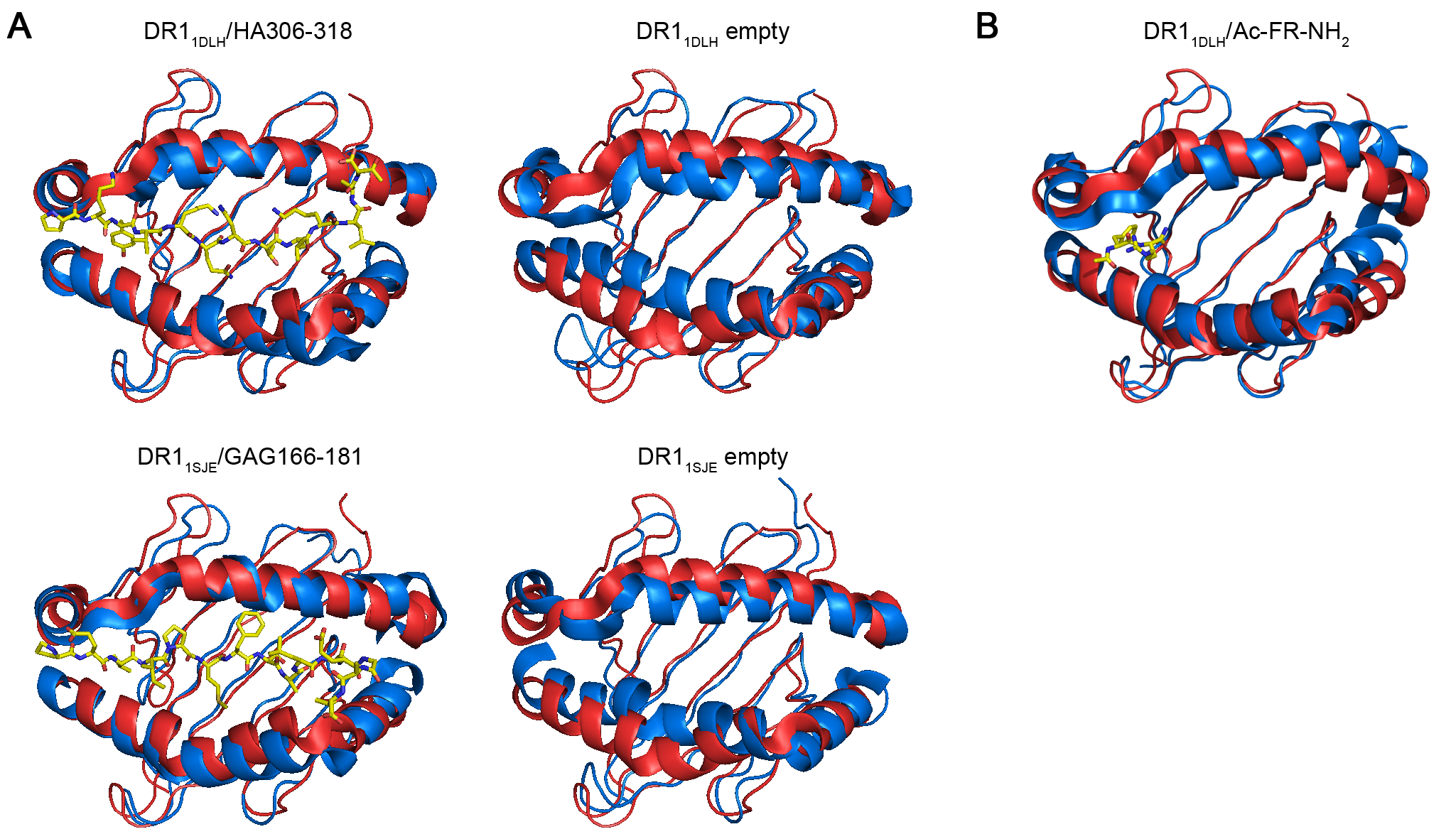

Supplement: Figure S1 — Antigen-binding site is closing in the absence of P1-occupation. The structure of the antigen-binding site of HLA-DR1 in representative frames of the MD simulations is shown. The starting structure is in red, the equilibrated end structure in blue. A, Comparison of peptide-loaded (left) and peptide-free (right) simulations of both crystal structures used in this study (1DLH upper panel, 1SJE, lower panel). B, Occupation of P1-pocket by the dipeptide leads to stabilization of the whole antigen-binding site during MD. (TIF) [file pone.0018662.s001.tif]

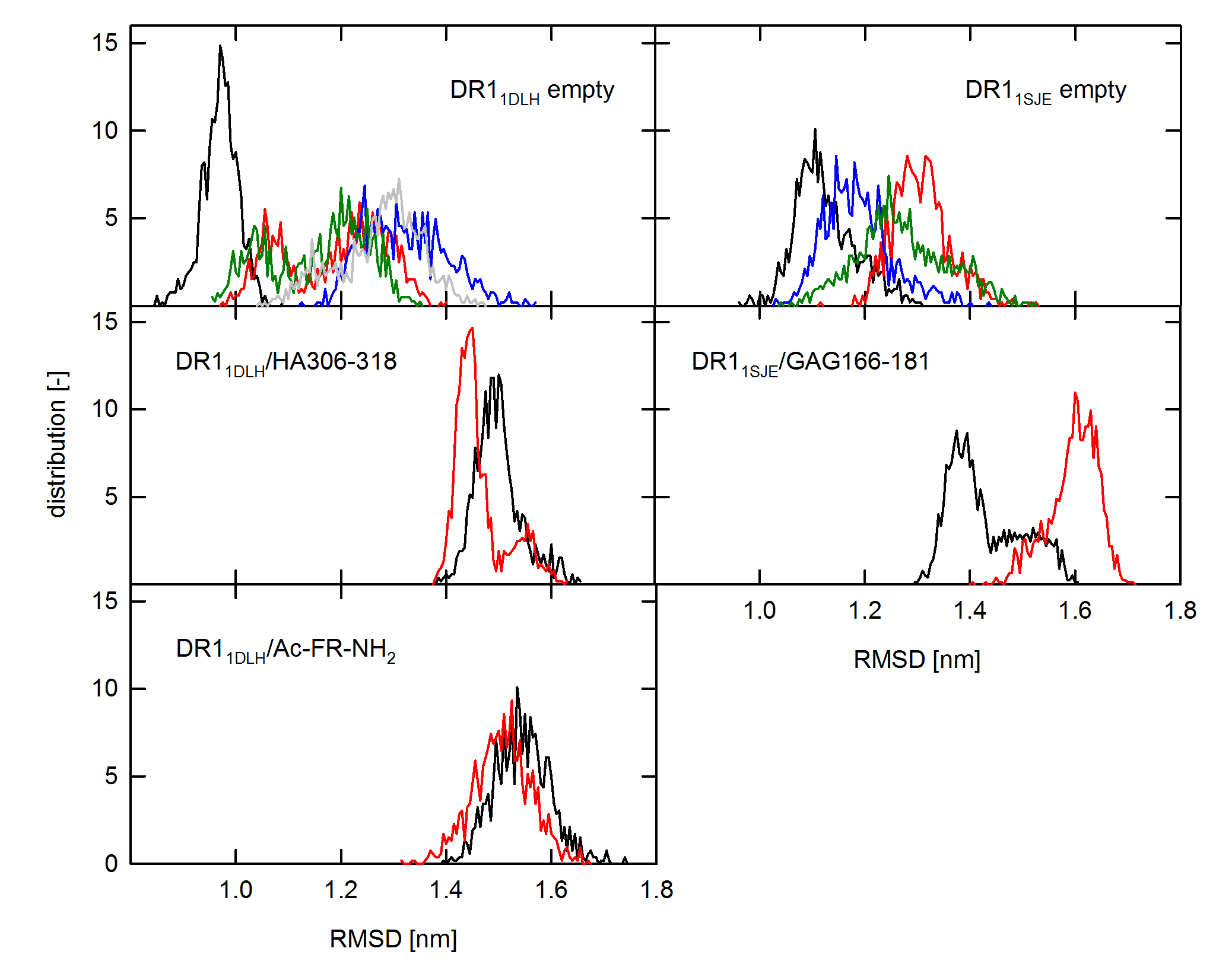

Supplement: Figure S2 — Distance of the helical regions flanking the P1-Pocket. The distance distribution for each individual MD simulation is shown. The distance was measured between the centers of mass of the α-helical regions flanking the P1-pocket (α51–61 and β73–85). The different starting structures are indicated in the graphs, the individual runs are shown in different colors. The distance distribution for the replicated runs is comparable. Only in one out of nine runs with empty HLA-DR1 a very narrow distance was reached (DR11DLHempty, black line, upper left panel). Visual inspection of the corresponding MD simulation reveals in this case a drastic inwards motion of the β-chain α-helix resulting in a collapsed antigen-binding site. The significance of this singular event, however, is not clear. (TIF) [file pone.0018662.s002.tif]

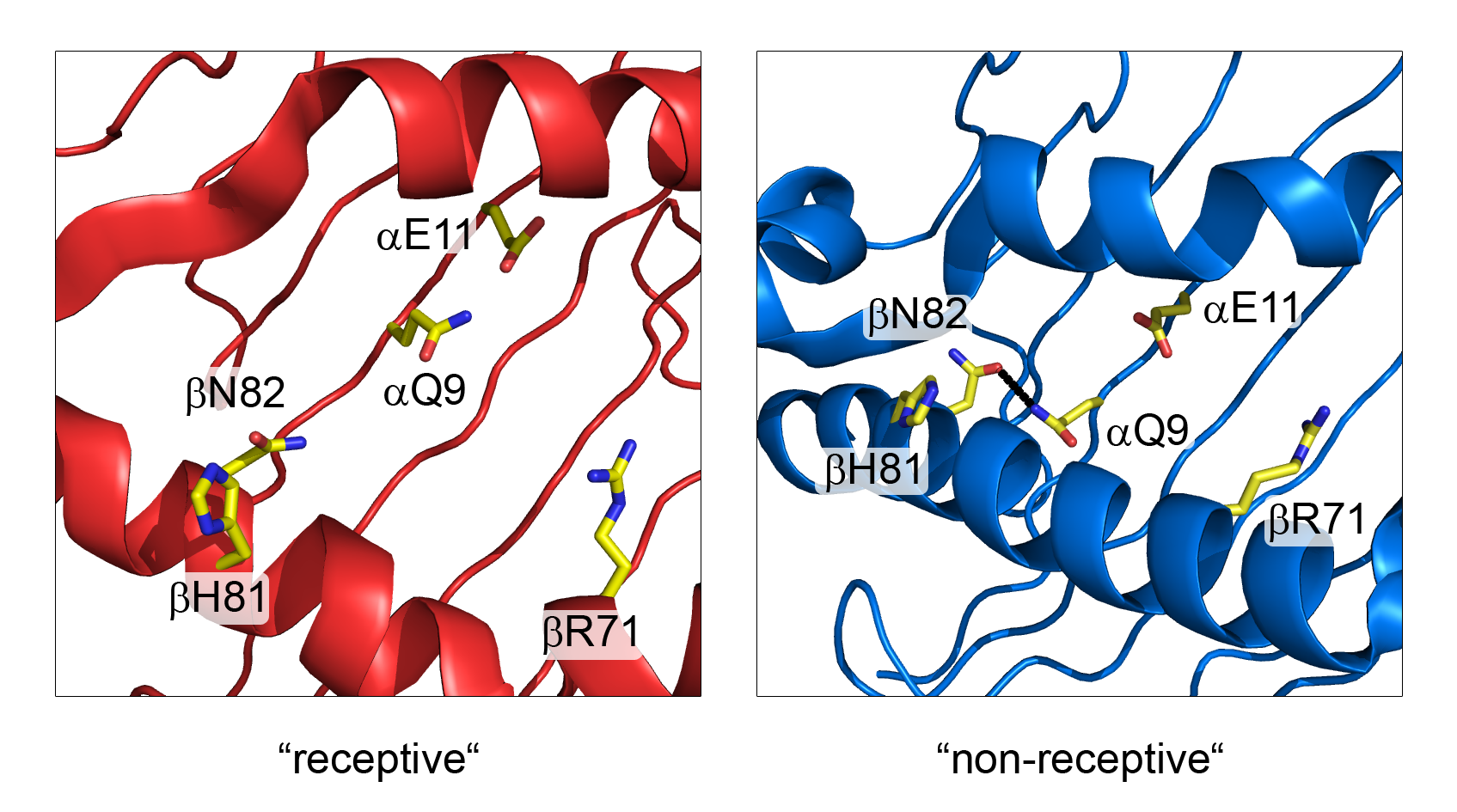

Supplement: Figure S3 — Position of residues used for introduction of point mutations to probe the putative locking mechanism. The amino acids are shown as yellow sticks. Left, open binding site at the beginning of the MD simulations. Right, closed binding site of the equilibrated closed structure of empty MHC II. αQ9 and βN82 are forming an H-bond (black dashed line). Residues βR71 and βH81 are apparently not involved in the stabilization of the non-receptive structure. (TIF) [file pone.0018662.s003.tif]
